# Dr. Golis's Treatise on the Hydrocephalus Acutus

**Published:** 1821-12-01

**Authors:** 


### Medico-Chirurgical Review,

AND

### JOURNAL OF MEDICAL SCIENCE.

(Analytical Series.)

"Perhaps that is nearly the perfection of good writing which effects for knowledge what the lens effects for the sun-beam—it condenses its brightness, in order to increase its force."—*Lacon.*

Vol. II.] DECEMBER 1, 1821. [No. 7.

### I.

*A Treatise on the Hydrocephalus Acutus, or inflammatory Water in the Head.* By Leopold Anthony Golis, Physician and Dissector to the Institute for the Sick Children of the Poor at Vienna. Translated from the German, by Robert Gooch, M. D. One volume, 8vo, pp. 280. London, 1821.

It is not amongst the least of the blessings of peace, that the scientific and literary, as well as the commercial, stores of Europe are opened, and their treasures diffused over the world at large. In language, however, which is the great medium of communication, there still exists a powerful check on its own operations—a defect which has not ceased to embarrass social intercourse from the "confusion of tongues," at the building of Babel, down to the present moment. Translation, the great remedy of this evil, is seldom rewarded in proportion to its merits: yet there is not a more praiseworthy undertaking than that of clothing, in vernacular language, the useful productions of a foreign soil—especially of a German soil, fertile as it is, in intellectual vegetation, but difficult of access over the frowning barriers of its rugged literature.

The work before us is characterized by Dr. Gooch, a gentleman whose competency of judgment will not be questioned, as "the best book he ever read on the acute hydrocephalus." The author, Dr. Golis, has been physician to an extensive institute for sick children, since the year 1793, enjoying great opportunities for observation, and possessing "practical talents of a very high order." Under these propitious circumstances, the reader will be prepared to expect something beyond the usual run of medical publications: and in this expectation we believe he will not be disappointed. True it is, as Dr. Gooch has justly observed, the work in question "like all books, has its weak parts;" but where are we to look for perfection—except in the sublime dicta of the infallible critic, or his imaginary BOARD of censors? Dr. Golis informs us that he has already opened 180 bodies of those who have died of hydrocephalus, in the presence of several physicians and surgeons, and that these dissections always confirmed the accuracy of his diagnosis. We confess that such an assertion surprized us not a little; for, according to this statement, the Doctor's diagnostic acumen was as great at the beginning, as it was at the end of his experience! But let that pass. It is only a way of talking, not very unusual among continental writers of the first respectability, though confined, in this country, to authors who seek fame or fortune through the medium of popular credulity, rather than the esteem of their brethren.

Dr. Golis divides hydrocephalus into the *hyper*-acute, acute, and chronic forms; denominating the first *wasserschlag*, or, in our language, "WATER-STROKE."

"The hydrocephalus *hyper*-acutus, apoplexia hydrocephalica, wasserschlag, literally water-stroke, is a *sudden* effusion of fluid within the brain, either occurring idiopathically, or the consequence of the repelled matter of a previous disease (defect of crisis,) or the consequence of obstructed evacuation from an excreting organ, from which death occurs in a few hours. To this belong all those depositions on the brain which arise from small-pox, measles, erysipelas, and other febrile eruptions; also those convulsions which follow the sudden cessation of chronic, or habitual discharges, the repulsion of chronic eruptions, as crusta lactea, tinea, discharges from the ears, and the like, or from diarrhœa, dysentery, general perspiration, when the same has been suddenly stopped without previous perceptible turgescence or inflammation. In all these cases of sudden death, there is found, on examining the bodies, an effusion of fluid in the head, for the most part in the ventricles of the brain itself." P. 6.

It appears, then, that in this form of the disease the stages of turgescence and of inflammation are wanting—a circumstance which distinguishes it from *acute* hydrocephalus. Here, too, the effused fluids are always found turbid, and accompanied with less coagulable lymph than in hyd. acut. It is almost needless to remark that the water-stroke runs such a rapid course as to give no time for remedies to produce any effect—it is therefore invariably fatal.

*Hydrocephalus Acutus.* In this form the effusion is always a consequence or sequela of previous turgescence and inflammation of the membranes or vessels of the brain, consisting in a collection of serum and coagulable lymph—the *former* in the ventricles or substance of the brain; the *latter*, like a membrane, filling the depths of the convolutions, and lining the walls of the ventricles as a preternatural coat; or covering the basis cranii, and hindering the absorption of effused fluid. The inflammatory nature of the complaint is inferred from the symptoms, which are those of encephalitis; from the influence of medicines; and from the appearances on dissection. From an attentive observation of nature, Dr. Golis divides this disease into four stages, which he thinks have distinguishable symptoms—viz. that of turgescence, inflammation, effusion, and paralysis.

*Turgescence.* Of the premonitory symptoms of the *turgescent* stage Dr. Golis draws a minute, and, we believe, faithful picture. We can only notice the prominent traits. Children grow indifferent to things that formerly amused them—become inactive, silent, irritable, disliking light and notice. The eye and countenance lose expression, the body plumpness, the bowels and kidneys activity. On getting out of bed the child complains of giddiness, and moves the hands towards the back of the head, with a whining tone. The pulse, though generally natural, will be found, on attentive observation, "to beat oftentimes a few beats weaker, and sometimes to intermit altogether"—the skin is without perspiration—the colour of the face changeable—the gait is tottering. Such are the premonitions in healthy children; and it must be confessed that very many of them precede other diseases than hydrocephalus acutus. Dr. Golis also acknowledges that, in feeble, irritable, scrofulous, and unhealthy children, this premonitory train of symptoms is generally overlooked, even by the most acute physicians.

"Indifference succeeding to increased sensibility and irritability; a constipated state after habitual looseness or diarrhœa; a scanty, unusually yellow urine, with or without sediment; dryness of the skin, which, previously, on the slightest exercise, even on eating and drinking, and particularly during sleep, perspired profusely; sleep without medicine often suddenly occurring in restless children; remarkable gravity and earnestness, which had never been previously noticed; these taken together, with the symptoms already mentioned, are the signs by which the turgescence of hydrocephalus may with great justice be suspected." 16.

In children of one, two, three, or four months, the premonitory symptoms are very equivocal, and require a careful consideration of all the symptoms in connexion.

The rarest mode of approach is what our author calls the *tumultuous;* where, for instance, a lively healthy child, after a sudden accession of languor, confusion, giddiness, head-ache, gastric irritability, full, hard, slow pulse, ocular sensibility, tinnitus aurium, &c. is suddenly seized with violent fever, and, generally, convulsions. If the practitioner now uses efficient means, effusion is arrested; but, "if he does not apply the necessary remedies with overwhelming power," effusion will take place, in one, two, or three days, "but most commonly in a few hours," and the child will be irrecoverably lost. The duration of the turgescent stage is frequently only a few hours; but often eight, ten, fourteen, or even more days.

*Inflammatory Period.* In this stage the symptoms of turgescence are merged in those of phrenitis. The little patient complains of severe head-ache, pressing upon the eyes—tension and shooting sensation in the nape of the neck—great restlessness—high sensibility of the eye to light, opening perfectly only in the dusk—heat of the head to the touch, particularly the forehead and nape—but the vessels of the surface are rarely turgid or red. In the tumultuous accession of the inflammatory period, there are convulsive tremblings of the eyes. The carotid pulsations are now evident, and there is a peculiar alteration of the physiognomy, in acute hydrocephalus, which our author considers to be pathognomonic. The nose is dry—the lips a faint dark red, and chapped—the tongue becomes covered with a white or brownish-yellow fur—thirst and appetite generally cease—vomiting usually occurs four or six times in the twenty-four hours—digestion ceases—food taken many days before is often passed undigested, with much slime, and a peculiar foul smell. The breath, at this period, has generally a bad odour—there is tenderness, on strong pressure, in the region of the stomach and liver—the belly, before tumid, now falls in, which our author considers as another pathognomonic sign of acute hydrocephalus, and the surest distinction between that and typhus. The bowels often remain obstinately constipated, in spite of purgatives—the stools are gluey, most commonly brown, sometimes yellowish-green—"only during the use of calomel green in all shades, and not very fetid." The urine is scanty, and often passed with pain—at the beginning turbid and white; but in the following stages of a bright yellow, with white, heavy, slimy deposit. Our author has not seen the lateritious sediment.

The sense of hearing now becomes acute—pains are felt in the belly as well as head—the nights are sleepless or disturbed—there is grinding of the teeth—starting and screaming out of sleep—languor of movements—inability to sit up without support, or without inducing nausea and vomiting. The pulse is now slow, unequal, and intermitting, except when the patient happens to be suddenly excited by a frightful dream or great pain in the head, when "the quickness of the pulse doubles in a moment." The pulse above described Dr. Golis considers pathognomonic of the second stage of hydrocephalus.

The skin, before tense, becomes flaccid—the patient commonly lies on one side, with the hand under the head—often shifts posture, or desires to be placed in the mother's lap, hoping, in vain, for relief by change of situation.

*Third Stage, or Period of Effusion.* The greater number of the above symptoms, after lasting from a few hours to two, four, six, or even more days, become exasperated. The patient can no longer sit up—the restless desire for change of posture ceases—he generally lies on his back, kicking up the bed-clothes with one or both feet, and often moving the hand to the head, mouth, and nostrils, the latter of which he picks till the blood comes forth. The tones of the voice are nasal, and the words indistinct—the little patients catch at imaginary objects, or pull themselves by the hair of the head, or pick their lips. All the senses, except that of hearing, become dull or annihilated—the focus of vision becomes dislocated—the pupils are dilated, and insensible even to strong light—vision itself is very indistinct, and often double—there is moaning and sighing—a gloomy earnestness is painted in the flushed countenance, with a threatening expression, broken by intervals of pitiable expression, and exhibiting a curious contrast of fierceness and patience, indicative of the inward distress.

Emaciation advances—the dry flabby skin hangs round the shrivelled limbs—the urine passes unconsciously—a stool seldom follows even a large dose of calomel, without a glyster, and is, for the most part, natural in consistence, soft, pap-like, or figured, brown, black, stinking. Diarrhœa, without purgatives, is of rare occurrence. When it takes place the stools are green, watery, slimy, and passed with pain. The pulse increases in irregularity and weakness—the respiration is more and more interrupted by sighs—the breath more fetid—in short, the soporose state passes into complete coma, and the last tragic scene approaches, before which the sufferers sometimes recover, for a short period, their consciousness, and are able to take food and drink, thus deceiving the physician and mother with momentary hopes too soon again to vanish. It is almost needless to say, that in this and the succeeding stage recovery is impossible.

*Fourth Stage, or Period of Paralysis.* The preceding or third stage, after lasting ten, fourteen, fifteen, twenty-one, seldom thirty days, terminates in convulsions, followed by palsy, generally of the right side, and often by cramp, which draws the head backwards and downwards, ceasing only with death. A violent fever takes place in this last stage, the ultimate exertion of nature, and a vain one, to remove the cause of death—the fluid effused on the brain. A hectic redness alternates with a deadly paleness on the disfigured countenance—the sight is gone—the pupil of the convulsed eye is greatly dilated, immoveable, insensible to the strongest light—the albuginea is blood-shot—the eye itself advances more out of its socket. The sense of hearing, hitherto morbidly acute, becomes gradually dull—swallowing is difficult, and, at length, impossible—the urine passes involuntarily—the evacuations by stool are few, being green, sometimes dark brown, slimy, but not fetid—the pulse becomes exceedingly quick, intermitting, irregular, weak, and almost imperceptible, with a corresponding state of the breathing—and thus, after nameless sufferings, from thirteen to twenty-four days, "the little sufferers, wasted to a skeleton, expire." 37.

*Diagnosis.* We have been very careful to convey to the reader all the leading and many of the minute features of Dr. Golis's symptomatology, in order that we might avoid, as much as possible, the diagnostic part of the volume, which contains numerous repetitions, and is uselessly minute, and we firmly believe, in many places, erroneous. We shall, however, introduce his diagnosis between hydrocephalus acutus and infantile remittent, or worm fever, entire; because we could not abbreviate it, and we consider it as by far the most important and practically useful portion of the section on diagnosis.

"1. The acute hydrocephalus has four stages, distinguished by a change of symptoms, seldom lasts less than thirteen, or more than one-and-twenty days, and if not cured at the beginning, always terminates in death. The worm-fever has no distinct stages, no regular change of symptoms, seldom terminates before the one-and-twentieth, often not till the thirtieth or fortieth day, and is often cured at every period of the disease.

"2. The acute hydrocephalus attacks strong, healthy children oftener than weak ones, and boys oftener than girls. The worm-fever attacks phlegmatic, over-fed, large-bellied, bad-complexioned children, and according to my experience, girls as often as boys.

"3. The acute hydrocephalus has no distinct remissions, and is never epidemic. The worm-fever commonly has remissions, and, though seldom, is sometimes epidemic.

"4. In the acute hydrocephalus there is, even in the beginning, a striking change of countenance and actions. In worm-fever the face is pale, and commonly swollen throughout the whole disease, and the conduct betrays sluggishness.

"5. In the second stage of the acute hydrocephalus, there is violent pain in the forehead, continuing during sleep, alternating with pains of the belly and stomach; likewise a sense of tension in the nape of the neck, hands, and feet. In the worm-fever there are pains in the head and belly, but they are dull, not much complained of, and not distinct in their seat.

"6. In the turgescence of the acute hydrocephalus the patients lose their appetite and thirst, go seldom to stool, and pass urine at first scanty and milk-white, which afterwards becomes yellow, and deposits a white, heavy sediment. In worm fever they eat greedily, drink thirstily, often pass large stools spontaneously, and often a great quantity of colourless urine.

"7. In the acute hydrocephalus the patients never sleep quietly, soundly, and refreshingly; they wake out of the sleep, which resembles a confused slumber, very easily, and feel after it weaker than they were before; they moan and whine in it only slightly, and if they sometimes cry out loud, it is with a tone expressing pain, and sighing they turn from side to side. In worm fever the sleep is always sound; we can scarcely wake the little patients; during sleep, they sometimes cry out vehemently, and not unfrequently jump completely out of bed.

"8. In the acute hydrocephalus the pulse is not quick till the fourth stage. In the first it is almost natural; in the second and third preternaturally slow. In the worm-fever it is quick throughout the whole of the disease, and seldom or never below the quickness natural to the patient's age.

"9. In the acute hydrocephalus the skin remains dry till the end of the stage of effusion. In worm-fever patients perspire after eating and drinking, and on every exacerbation of fever.

"10. In the acute hydrocephalus the patients often fall into deep musing fits, out of which they wake with a sigh. In worm-fever, dullness, tedium, stupidity, are painted on their countenance.

"11. In the acute hydrocephalus, in the stage of turgescence, the patient's step is without steadiness, weak, uplifted as if going up a step. In the worm-fever the walk is slow, but neither weak, nor unsteady, nor uplifted.

"12. In the acute hydrocephalus the patients in the imflammatory period are continually changing their posture in bed: in the moment of effusion they lie for a long time on one side, the lower hand under the head, the upper in automatic motion near the head.

In the worm-fever the patients lie still in bed, often cry out with fear, or spring out of bed in their sleep, or sometimes wake out of sleep, in which they lie across the bed, or with the head where the feet should be.

"13. In the acute hydrocephalus the sight is over-sensible in the inflammatory period, dim in the stage of effusion, and blind in that of palsy. In the worm-fever the patients never complain of strong light, nor are they perfectly blind at the approach of death.

"14. In the acute hydrocephalus the hearing is preternaturally quick till the last stage of the disease. In the worm-fever they are slow of hearing, particularly towards the *end of* the disease.

"15. In the acute hydrocephalus the nasal mucus is diminished in the stage of turgescence, and entirely stopped in the following stages, the patients gradually losing their smell: there is no violent itching of the nose. In the worm-fever the inner surface of the nose is moist, the smell acute, and there is always an intolerable itching of the nose.

"16. In the acute hydrocephalus the vomiting which indicates the second stage occurs in every case. In the worm-fever it is an accidental symptom, which is remarked only when worms have made their way into the stomach.

"17. In the acute hydrocephalus, what is rejected from the stomach has a peculiar foul smell. In the worm-fever it has not.

"18. In the acute hydrocephalus the respiration is natural in the second and third stages. In the whole progress of the worm-fever it is accelerated during the febrile exacerbations.

"19. In the acute hydrocephalus the bowels are constipated, particularly in the three last stages. In the worm-fever, a ready action of the bowels, even diarrhœa, from slight laxatives, are common appearances.

"20. In the acute hydrocephalus the urine is very scanty, and passed in the two last stages unconsciously; in appearance, it is natural in the stage of turgescence: in the inflammatory stage, white and turbid, with or without white sediment; in the two last stages it is of a high yellow, with a white slimy, heavy sediment. In the worm-fever, the urine is sometimes turbid, whey-like, and in small quantity; sometimes as clear as water, and in large quantity; its dirty white sediment is not so heavy as that in hydrocephalus; the first is passed with difficulty, the last without, but commonly not insensibly, in bed.

"21. In the acute hydrocephalus the head feels warmer than the regions of the stomach and liver, and these again warmer than the other parts of the body; in the paralytic stage the unpalsied side is warmer than the palsied. In the worm-fever most commonly the belly only is hotter than the other parts of the body.

"22. In the acute hydrocephalus the plumpest children waste rapidly; their belly, if it had been large, falls in in a few days, without proportionate evacuations; the sound of flatulence is seldom or never heard. In the worm-fever there is no wasting of the body; the belly does not shrink; flatus rumbles in the bowels, and passes audibly.

23. In the acute hydrocephalus palsy of one side, or of some part, often with cramp of the spine and general convulsions occur in the fourth stage, terminating in a few days with death. In worm-fever convulsions may occur without palsy of any part, at any period of the disease: if any palsy is remarked, it is transient.

"24. In the acute hydrocephalus towards the approach of death, hectic redness of the cheek alternates with deadly paleness, and the tips of the fingers are red: these appearances are never observed on the approach of death in worm-fever.

"25. In the acute hydrocephalus towards the latter end, there is a peculiar eruption about the mouth, and on many parts of the body; this characteristic eruption is entirely absent in worm-fever; frequently there appears instead a miliary eruption, the common accompaniment of gastric diseases.

"26. In the acute hydrocephalus the countenance expresses inward suffering. In the worm-fever the countenance is without any expression." 54.

The diagnosis between acute hydrocephalus and typhus occupies between fourteen and fifteen pages, and appears to us quite superfluous—in this country, at least, where typhus is viewed in a much more restricted light than on the Continent. The diagnosis between water-stroke and "masked fatal intermittent," we shall also pass over, especially as our author informs us that the appearance of the latter disease under the mask of the former "is in the highest degree rare"—in fact, not having been seen once by Dr. G. in twenty years' experience.

*Etiology*. Dr. Golis is sufficiently minute in his description of the *predisposing* causes of this disease; but does not offer much which we deem it necessary to notice in this article. Among the *exciting* causes he lays great stress on cold applied to the heads of new-born infants, the consequences of which are, in his experience, "internal inflammations in the head, which occasion the effusion of lymph and of serum in the cavities of the head and brain, terminating in death, which appearances are merely called convulsions." 79. But the most important cause of all, according to Dr. Golis, in large, healthy, and lively children, who have begun to run, romp, and jump about, is mechanical *agitation* of the brain, nearly the major part of the sufferers whom he has had occasion to treat having come by the disease in this way. The next exciting cause, in order of importance, "is the sudden drying-up of discharges from ulcers and moist eruptions on various parts of the surface of the body"—also the disturbance of the febrile exanthemata, as measles, variola, scarlatina, &c. External inflammations of the head and neck, and all kinds of erysipelas which occasion increased turgescence in the head, or propagate phlogosis to the cerebral coverings, are liable to produce hydrocephalus, especially the hyper-acute form, or water-stroke, falsely called convulsions. Indeed, vehement inflammations of remote parts or organs will do the same, whether by impeding the circulation and causing a determination to the brain, or by means of that sympathetic connexion which the sensorium must have with all parts of the body. Violent vomiting, the incautious use of belladonna in hooping-cough, abuse of fermented liquors, insolation, suddenly suppressed diarrhœas and dysenteries, have all produced, in the experience of our author and many others, hydrocephalus acutus.

*Terminations.* The water-stroke ends fatally—acute hydrocephalus is, for the most part, fatal; but sometimes terminates in recovery, or in some other disease, as blindness, deafness, idiocy, epilepsy, hemiplegia, marasmus, chronic hydrocephalus. Those patients who are cured, can be saved only in the stages of turgescence and inflammation. Death seldom takes place earlier than in the fourth stage, unless where the disease begins with tumultuous symptoms. The duration of the disease will easily be collected from the symptomatology.

*History.* Dr. Golis contends, and we think with reason, that the disease was known to Hippocrates, as certified by the following passage.

" 'Aqua, si in cerebro suborta fuerit, dolor acutus sinciput et tempora interdumque alias capitis partes detinet, subindeque rigor et febris, oculorum regiones dolor occupat, iique caligant; pupilla scinditur, et ex uno duo sibi cernere homines videntur, et si quis surrexit, tenebræ ipsum prehendunt, neque ventum neque solem sustinent, aures tinniunt, salivam et pituitam vomitione refundit, quandoque etiam cibos, &c.' " 87.

The first, however, who rightly collected the symptoms of acute hydrocephalus, and wrote a separate treatise on the disease, was unquestionably our own countryman, Robert Whytt; since whose time, many dead and living authors have published on the same subject.

A more interesting point is, whether the disease is becoming more frequent than formerly? Dr. Golis is convinced, from wide and accurate observation, that this, as well as croup and other inflammatory effusions have, for the last ten years, been much more frequent than at former periods. The *causes* of this increase of frequency are very difficult to be ascertained. Dr. Golis inclines to the opinion of many physicians, that—"the present great frequency of this disease is to be sought for in the less frequent eruptions on the heads of children; believing, with them, that the lymphatic system has suffered a great revolution, since which, the achores have gradually vanished, and the diseases of effusion have become more frequent in children." We have not the least doubt but that the various tribes of human afflictions have been perpetually, and will for ever continue to be, undergoing revolutions in their nature, and consequently in their treatment—events that will never permit the pathologist and physician to enjoy a holiday in their labours; for, as it was in the beginning, we believe it will always continue—"sufficient for the day will be the evil thereof."

Our brethren of the Emerald Isle will be a little astonished to learn that some of the continental physicians attribute the increase of hydrocephalus to—the eating of *potatoes!* If we may judge of the material, by the intellectual, *effusions* of Irish brains, we should not be very apt to characterize them as a *watry-headed* race.

A more rational cause of the disease under consideration, has been sought in the modern physical education of children, by which they are accustomed to take spiced foods, and spirituous drinks, in the first months of their existence. Dr. Golis imagines there may be something in this; but attaches far greater importance to "the rarity and almost disappearance of the achores."

*Prognosis.* Under this head we find many interesting remarks, with some unnecessary repetitions. The water-stroke, we have seen, is always fatal, and the acute hydrocephalus, when it has arrived at the third stage (effusion)—perhaps when it has gone beyond the *beginning* of the inflammatory stage, is also beyond the powers of our art.

"The more sudden the attack, and the more violent the symptoms, so much the more rapidly the stages follow one another, and so much shorter is the duration of the disease. On the contrary, the more gradually it approaches, the more mild and few the symptoms are, the less they are distinct, the slower is the transition out of one stage into the other, and the later does death terminate the sufferings of the patient." 95.

"If, in the stage of turgescence, or inflammation, by the use of antiphlogistic remedies, as calomel, and external and internal evacuants and counter-irritants, the hydrocephalic symptoms are entirely removed, and do not return for two or three days, during a perseverance in the remedies, there is hope of recovery; but if, during the above-mentioned days, there is a recurrence of irregular pulse, pain in the head, and vomiting, then between the thirteenth and seventeenth days, seldom later, death unavoidably follows." 97.

When, in the progress of the second stage, after blood-letting, calomel, &c. a general steaming perspiration breaks out during sleep, and continues several hours, it is a good omen, and arrests the effusion of lymph or serum in the cavities of the cranium. Partial and general sweats, in the fourth, or paralytic stage, are the near forerunners of death.

Even when the treatment is conducted in the most prompt and judicious manner, and attended with the most striking amendment, we are never sure of success. Dr. Golis has often seen the distressing head-ache, the violent vomiting, the optic sensibility, and other symptoms, vanish—the slow intermitting pulse become natural—the disturbed vital functions fall into order—the appetite for food and drink return—and yet, in 24 or 48 hours, death ensue.

*Pathology.* Dr. Golis makes therapeia precede pathology, which we think is a very unnatural arrangement, and we take the liberty therefore to reverse it. We shall pass over what others have observed, (particularly as we offered an extended article on this subject, in the 8th number of our last series, for April 1820,) in order to present the result of our author's own observations.

Dr. Golis found the brain firmer, and the blood-vessels less turgid, in the water-stroke than in the acute hydrocephalus:—the turgescence of vessels and consistence of medullary matter being always in exact relation to the duration of the disease—that is, "the shorter the disease, so much the firmer and more consistent was the brain, and so much less enlarged and less turgid were its blood-vessels, and *vice versa.*" The plexus choroides he always found pale, and, in chronic hydrocephalus, disorganized and diminished. In acute hydrocephalus Dr. Golis generally found the sutures a little separated—not so in the water-stroke. In a few cases, where all the symptoms denoted effusion, no water, or but a very insignificant quantity, was found on dissection. But, as we stated in our article on hydrocephalus, in the 8th number of our quarterly series, there was turgescence of the blood-vessels "which, by their volume, like real extravasation, compressed the brain." *Golis,* p. 186. In one case, our author believes there was a kind of effusion into the substance of the brain, which swelled up as soon as the calvarium was removed, and from which a quantity of serum oozed, when it was cut in slices. In respect to the quantity and quality of the hydrocephalic effusion, there is great variety of statements by authors.

"I found in the water-stroke from 2 to 4, or 6 ounces of turbid fluid; in the acute hydrocephalus, for the most part, the same quantity, seldom a greater, and always clear; and in the chronic hydrocephalus, 1, 4, 6, even 8 pounds of equally clear fluid." 189.

"Likewise I saw, in all my dissections, in the water-stroke, and in the acute hydrocephalus, as did Morgagni and many others, the choroid plexus always pale and bloodless, and in chronic internal hydrocephalus, the organization of the brain, for the most part, destroyed." 190.

On this subject, Dr. Golis observes, "Dr. Baillie's anatomical remarks are excellent." With the latter illustrious English pathologist, Dr. Golis has found hydatids in acute hydrocephalus. In a very few cases Dr. G. found the effused water mixed with blood; but then there were complications, as inflammation or caries of the ears, &c.

Our readers will perceive that Dr. Golis merely states the *appearances* on dissection, but does not go into the *modus agendi* of the causes, or into any theory of the disease. We now, therefore, come to therapeutics.

*Treatment of the Acute Hydrocephalus.* It is much more easy to enumerate the proper remedies in this dangerous disease, than to point out the proper time and mode of their exhibition. Hic labor, hoc opus.

1. *Stage of Turgescence.* In this, as in all other diseases, it is necessary to use our best endeavours to discover the *causes* of the malady, and then to study the individual constitution and idiosyncrasies of the patient.

"Thus it is very important to know whether any eruptions have been repelled; old ulcers suddenly healed; habitual discharges from the ears and other parts have been suddenly stopped; whether the liver is diseased, or the mesenteric glands; whether narcotics, or intoxicating spirits have been taken; perspiration has been suddenly suppressed; the brain has suffered any mechanical agitation. Each of these must be accurately considered; the plan of treatment must be directed, not only generally against the disease, but specially, according to the exciting cause, and the constitution of the patient." P. 104.

Finding, then, the symptoms of turgescence, as described in the proper place, and having investigated the cause and constitution, the patient is to be placed in a roomy chamber, screened from strong light, and on a bed slightly inclined. He should be cautiously raised and laid down, as all quick and rough movements increase the giddiness and uneasiness of the head. The temperature of the room should be rather cool than warm—the head a little raised and uncovered—the bed-clothes light—a familiar nurse employed to prevent irritation; and no force used in the exhibition of medicines, if possibly avoided. To prevent turgescence from passing into inflammation, the remedies are, calomel, antiphlogistics, evacuants, and counter-irritants.

"Of all the medicines which have been highly praised for the acute hydrocephalus, calomel is the most efficacious; in the turgescence, and at the beginning of the inflammatory stage, I may almost call it a specific: it excites, as it were, an abdominal or intestinal ptyalism, loosens the coagulating power of the lymph, and lessens, by the action which it excites in the alimentary canal, the orgasm in the head; awakes more activity in the ends of the serous vessels, by which absorption is increased, and, according to my experience, makes all other purgatives, for the most part, needless, and only in cases of very diminished irritability in the alimentary canal, or in very great collections in the same, is an addition of jalap necessary." P. 108.

Our author observes that the dose and continuance of the medicine do *not* depend on the age, but the constitution of the patient, and the irritability of the alimentary canal.

"Children of one year and under, bear, in general, a far larger dose, (for example, eight or ten grains of calomel in the twenty-four hours,) without producing diarrhœa, colic pains, swelling of the salivary glands, than children of four, five, six, or eight years, who will scarcely take three or four grains before they begin to complain of pains in the belly. I have never seen the use of calomel produce salivation within a few days, as Wilmer and Perkins have; and a London physician, who has written in the London Medical Journal for 1781: with Mier and Graham, I have seldom seen this appearance at all, and then only late, after long perseverance in large doses." P. 110.

In children of from one to five months, a quarter of a grain—from six months to one or two years, half a grain of calomel may be given internally every two hours, until it discharges green slimy stools four or six times—"but not purging stools." In habitually costive children "it is often necessary to give the dose of calomel every hour, and in a high degree of insensibility of the alimentary canal a few grains of *roasted* jalap must be mixed with the quicksilver, in order to produce the wished-for effect." Dr. G. found that the *roasted* jalap did not, like the *raw*, excite vomiting, which is to be carefully avoided—neither does it gripe. If the calomel produce sharp colic pains, it should be left off till they cease, or the diarrhœa (if any) subsides—then to be resumed at longer intervals, "as three or four hours, in half-grain doses, until the above effects, purging and colic, follow." In this way Dr. Golis perseveres in the use of the remedy as long as the symptoms require. The external use of mercury, our author thinks, is too slow in its effects, to be trusted to in such a disease. Dr. Golis does not agree with Whytt, Odier, Quin, Wilmer, Leib, and others, in the propriety of large doses of calomel to children in this complaint. He has seen inflammation of the intestines brought on by such practice. In this injunction we entirely agree with Dr. Golis.

"If this highly-praised remedy does not always produce the wished-for effect, the bad preparation of the medicine is not unfrequently the cause; the only contra-indications which can forbid the use of the calomel, are violent pains in the abdomen, an inflammatory state of the stomach and intestines, and weakening diarrhœa: but as, in the many hundred patients whom I have attended for the acute hydrocephalus, I have never seen inflammation of the belly, violent pains, or diarrhœa, but instead of the last, commonly constipation; so I have never neglected, when I was called during those moments, when a cure could be hoped for, to employ this remedy. Yet, should the above-mentioned contra-indications occur, no practical physician would prescribe it under such circumstances." 115.

*Emollients.* Next in importance to mercury Dr. Golis ranks *emollients*, or what we term in this country *diluents*. They should not, however, possess any acescent quality, on account of the calomel. Vegetable infusions or light decoctions, as of marsh-mallow, barley—or merely solutions of gum arabic, with or without nitre, all having the temperature of the chamber, except at those times when it is desirable to promote diaphoresis—then they may be lukewarm. Gum arabic emulsion, our author asserts, alleviates the distressing head-ache sooner than any other medicine. Digitalis, sudorifics, diuretics, and tonics, are all improper in the stage of turgescence.

As *external* means, blood-letting—cold applications to the head—stimulating pediluvia—blisters—mercurial and antimonial frictions, and enemata, are the principal. General blood-letting is much less frequently necessary than local.

"According to my experience, blood-lettings are fruitless in the water-stroke, pernicious in chronic hydrocephalus, and only in the acute hydrocephalus, at determinate moments, and under certain circumstances, efficacious and necessary." 119.

Cold applications to the head, "whilst internally the calomel has produced a derivation and reaction in the alimentary canal, are of striking efficacy." Tepid baths or semicupia are dangerous. Pediluvia with mustard, &c. are all that can be ventured on in this stage. For blisters, the calves of the legs are the best place of application. From mercurial frictions there is little to be expected for the removal of the turgescent stage itself.

"Yet this is the time for employing them, if we expect from them any assistance in the subsequent stages of this disease, in case it passes into them. The occiput and nape of the neck, and the thighs, are the places to which they should be applied. If the mercurial ointment (unguentum Neapolitanum) is employed, a whole ounce must be rubbed in in four-and-twenty hours, that is, a drachm every three hours. If calomel is chosen instead, four or six grains in some vehicle may be rubbed into the same parts. These frictions may be employed at the same time with the internal use of calomel, and may be continued for twenty-four or thirty-six hours." 123.

The same observations apply to tartar emetic ointment, that were made on the unguentum hydrargyri.

Children at the breast, or reared artificially, should use the same diet as before, but sparingly.

2. *Inflammatory Stage.* In this stage blood-letting, of course, holds the first rank.

"In healthy, active, strong, plethoric children, in the first six months of life, particularly after violent agitation of the brain, and in an inflammatory season, two, three, and in pressing cases of violent tumultuous accession of this stage, four ounces of blood may be drawn; from six months to one year, three, four, even five ounces may be taken at once with great advantage to the patient; in the second, third, and fourth year, the violence of the symptoms often demands a blood-letting of four, five, or six ounces, and in the later years of childhood, a still greater, which, according to circumstances, sometimes requires to be repeated." 128.

The above may serve as a kind of medium, the quantity, in each case, being always determined by the actual attendant at the bed-side of the patient. Long experience has taught Dr. Golis that, when the first bleeding was sufficiently copious, a second was seldom required—repeated small bleedings never answering well. Those cases of hydrocephalus which are excited sympathetically, occur symptomatically or metastatically, and in cachectic individuals, do not bear bleeding well, and in them it must be used with the greatest caution. As, in many infants, there is difficulty in drawing blood from a vein, leeches, in sufficient number, to the temples or behind the ears, are little inferior to general blood-letting, and may, in most cases, be preferred. At a more advanced period of life, however, and in cases of pressing danger, general blood-letting must be employed. In drawing blood the practitioner must pay the strictest attention to its effects on the pulse and the pain in the head.

"As long as the former continues irregular, and not very weak, as long as it does not return to the regularity of a natural, or a febrile pulse, and as long as a diminution of the vehement characteristic pains of the head, or a state of weakness does not take place, the blood may be allowed to flow." 134.

"At the same time at which the requisite blood-lettings are undertaken, internally the antiphlogistic remedies, and with them the calomel must be carefully administered to the patient; the former must be given luke-warm, liberally, and at short intervals; but in little infants, in doses proportionate to their age; and the latter, as I have already said of the treatment of turgescence, must be persevered in until colic pains occur, and several green stools follow. Only I must remark, that the calomel, in this stage of the disease, even in large doses, will often be given twenty-four hours without producing any stool, and that the patient will often have taken ten, twelve, fourteen, and even more grains, before a stool is procured. In these cases, as I have already said, some grains of roasted jalap should be mixed with the calomel; besides which, glysters of camomile tea with soap, honey, salt, sugar, and such like, should be employed, in order to hasten the action of the calomel, which (I now repeat once for all) is not indicated later than in this stage of the acute hydrocephalus, nor can contribute to the cure of this disease." P. 136.

In this, as in the turgescent stage, diluents, of course, are proper, and with them may be conjoined gentle diuretics, particularly the infusum digitalis. Sudorifics may now also be exhibited.

On digitalis Dr. Golis makes many interesting remarks. He considers that this medicine is by no means so efficacious here, as in acute hydrothorax after scarlet fever.

"Yet in the stage of turgescence and inflammation, in connexion with calomel and antiphlogistics, after suitable blood-lettings, where, with diminished power, there prevails an increased sensibility of the arterial system, it is very efficacious, as these combined remedies produce a powerful revulsion, by action on the alimentary canal and the urinary passages, occasioning determination *from* the head, and increasing the evacuations by stool and urine." 139.

In Dr. G's experience the medicine had little effect on the urine, and scarcely any on the pulse.

In the inflammatory stage, cold applications to the head, mustard cataplasms to the feet, blisters, and glysters, are equally proper as in the turgescent. The foot-bath is generally productive of inconvenience. Frictions with mercurial ointment and emetic tartar are useless in this stage, as their effects come too late. Blisters, in general, should not be applied till after blood-letting. The most usual positions are, to the occiput, the nape of the neck, or between the shoulders; but Dr. G. justly observes, that their operation, in these places, is often interfered with by the cold applications to the head, or the blood oozing from leech-bites. The belly and præcordia, "because of the great sympathy between them and the head," are the fittest places, Dr. G. thinks, for large or small blisters. The calves of the legs, the thigh, and the upper arm, are also proper situations for vesicatories.

If the physician have been fortunate in preventing or arresting the hydrocephalic effusion, he must maturely deliberate on what part of the treatment he is first to remit or omit—and what medicines to continue, in order to prevent relapse. The parts excoriated by the blisters, should generally be kept discharging for some time during the convalescence, to secure perfect recovery.

"In the after-cure, or treatment of the debility which the remedies have occasioned, bark, valerian, arnica, camphor, musk, castor, deserve the preference before the other medicines of this class; and in great morbid irritability of the arterial system the digitalis, in combination with one of the above-mentioned strengthening remedies, is the most effectual medicine for rapidly removing this morbid state." P. 158.

Dr. Golis begs to draw the attention of practitioners to that state of the patient "in which the symptoms of inflammation and of effusion seem mingled with one another." Under such circumstances, the same treatment as in the inflammatory stage, would, of course, be most proper, as the mistake, if any, would be on the safe side.

Of an operation to remove the water of *acute* hydrocephalus, we need hardly say any thing. In the *chronic* form of the disease, there is more feasability in the proposal, and we shall allude to the cases of Dr. Vose and some others in this country, in the proper place.

Dr. Golis has made a few observations on the *palliative* treatment in the two last, or fatal stages of the disease; but in these we do not see any thing particularly worthy of notice. The following passage on prophylaxis we shall extract.

"The prophylaxis consists in a suitable diet, in the preservation of perspiration, of the action of the bowels, of the secretion of urine, of the free circulation of blood in all parts of the body, of a good appetite and digestion, in a careful prevention of every thing which can increase the determination of blood towards the head, as violent and frequent movements of the body, agitation of the brain, abuse of spirituous drinks, spiced, heating food, overloading of the stomach, collections in the abdomen, constipation, and such like; further, in a gradual hardening of the body, by moderate exercise, by gradually leaving off warm clothing, and inuring to all temperatures of weather, but always with regard to the peculiar constitution of the child, to the mode of rearing which has already been employed, and to its age. Constipation should be prevented by gentle, not drastic purgatives, especially by small doses of calomel, mixed with a few grains of rhubarb, or by change of diet. Our forefathers, who cautiously, from time to time, gave children eccoprotics, may have contributed much to the prevention of this disease." 179.

Dr. Golis has seen some fatal instances of symptomatic hydrocephalus from inflammation of the bladder, brought on by imprudent retention of the urine during play. Parents should also be warned of the bad effects of tight dresses, immoderate eating, and violent agitations of body or mind. Nasal hæmorrhages, a common and beneficial occurrence in children, should not be interfered with, unless profuse. In all hurts and accidents in children, the consequences should be guarded against by blood-letting and antiphlogistic measures. Lastly, when a practitioner is called to a case, where the symptoms are ambiguous, and he "cannot determine with certainty whether they indicate acute hydrocephalus, or some other disease which resembles it," "let him apply those means which are serviceable in the turgescence of the acute hydrocephalus, and which can do no harm in any other resembling disease." This we believe is very judicious advice.

We have now presented the reader a comprehensive, and we hope clear, analysis of the historic, didactic, and preceptive portions of the volume: the remainder (about 80 pages) being occupied with cases in illustration, and formulæ of medicines. We do not consider it necessary to attempt any delineation of such a multiplicity of examples, especially as many of them are but very imperfectly or obscurely sketched. We shall therefore introduce a single case, taken almost at random, as a specimen.

" Henry A, four years old, vaccinated, strong, lively, and well nourished, heated himself by violent running in a spacious garden. Covered with perspiration he sat down with bare head and breast, and back covered only with his shirt, in a pouring rain, till he was wet through. The next morning he complained of weight in the head, tension at the nape of the neck, transient lancinating pains in the forehead, feebleness, absence of thirst and appetite, and slight fever, in which, however, the pulse was at the natural quickness and fullness; yet I already remarked irregularity in the beats, as some were hardly to be felt and others were omitted. He was constipated, had scanty though natural urine, the skin was dry to the feel. My first care was to restore perspiration, from which I expected much good. In the second night after this exposure to cold, the fever became greater, and, at the same time, the above-mentioned symptoms more violent; a remarkable remission of the fever followed towards noon of the third day. Emollientia, with radix sambuci, and ammonia acetata with syrup, were the medicines which he took; and mustard cataplasms to the feet, and glysters, which operated, were the external remedies which I employed. I expressed to the parents of the child, my fear of the acute hydrocephalus, and proposed blood-letting, at which they were more terrified than at the danger to which their child was exposed, because a surgeon, a stiff Brunonian without principles, had related some horrible stories about blood-letting, and prophesied the worst consequences. In a *consilium* with a learned physician and this surgeon, the disease was stated to be an intermittent fever, because at this time intermittents reigned epidemically in and about Vienna, and in spite of my remonstrances, Peruvian bark was ordered, which the parents with great readiness administered to their child. But the results verified my prognosis; for the inflammatory period with severe pains in the head, and all the symptoms which accompany this stage of acute hydrocephalus, shewed themselves. A second *consilium* with true practical physicians, attached to no system, and intimately acquainted with this form of disease, was now called, but too late. All the means employed which earlier would certainly have hindered the effusion, were no longer capable of arresting the progress of the disease. Insensibility came on, followed after six days by palsy with the most violent symptoms, and at the end of eight-and-forty days from that time, his sufferings ended.

"*Dissection.* This was attended by the physician who in the first consultation proposed the bark, and was performed by the surgeon. The blood-vessels of the covering of the cranium were turgid; the bared bones of the cranium were blue, the sutures were separated from one another by a line, and the interval was filled by a bloody extravasation. The blood-vessels of the membranes and of the brain itself were uncommonly large, and turgid with blood, as were also the sinuses, in which cruor and much lymph floated in the serum. Between the pia mater and the brain, which was firm and elastic, I met with much coagulable lymph. On the corpus callosum lay the same, about as thick as the back of a knife; and equally thick, at the basis of the cranium, where it enveloped the vessels and nerves. The ventricles, in which more than six ounces of clear water were contained, were lined by the same, through all their length and incurvations. The plexus choroides was very pale, and wholly covered with lymph. The pituitary gland was in its natural state, but covered with lymph; the septum of the ventricles was broken through; the white substance of the brain was of a reddish colour; the viscera of the thorax and abdomen were perfectly healthy. The incredulous physician began, after this, to believe in the acute hydrocephalus. Whether the surgeon, who soon after went to Russia, was converted, I know not." P. 230.

Fortunately these "stiff Brunonians without principles" are now rather scarce in this country, where physicians attend more to practical indications collected at the bedside of sickness, than to physical and metaphysical speculations engendered in the libraries of the learned.

In closing this analysis, we have no hesitation in stating it as our opinion, that the medical profession of this country is much indebted to Dr. Gooch, for introducing to their acquaintance an illustrious stranger, whose treatise reflects credit on the age and country in which it was produced. That Dr. Gooch's translation is sometimes rather stiff and literal, must be acknowledged—nor can it be said that, "materiam superabat opus." But where the *matter* is so excellent, the *manner* is of very subordinate consideration.

The experienced reader will readily perceive that Dr. Golis's description of the disease sufficiently proves that climate, education, and diet, often modify the features and course of maladies, and consequently that his symptomatology, *in some particulars*, does not exactly apply to hydrocephalus, as usually seen in Great Britain. Still, however, the general traits, and frequently the minutest shades of this insidious disease, are so evidently and so faithfully copied from Nature, that they will be read with interest, and remembered with advantage by practitioners of all ranks, ages, and countries. We shall look forward with much anxiety for the appearance of Dr. Golis's promised Treatise on *Chronic* Hydrocephalus, and hope Dr. Gooch will lose no time in presenting his countrymen with a version of the forthcoming essay.

